# Electrocatalytic hydrogen evolution with gallium hydride and ligand-centered reduction†
†Electronic supplementary information (ESI) available: Crystallographic data as CIF files; Fig. S1–S34; and Table S1. CCDC 1855035–1855038. For ESI and crystallographic data in CIF or other electronic format see DOI: 10.1039/c8sc05247f


**DOI:** 10.1039/c8sc05247f

**Published:** 2018-12-27

**Authors:** Ni Wang, Haitao Lei, Zongyao Zhang, Jianfeng Li, Wei Zhang, Rui Cao

**Affiliations:** a Key Laboratory of Applied Surface and Colloid Chemistry , Ministry of Education , School of Chemistry and Chemical Engineering , Shaanxi Normal University , Xi'an 710119 , China . Email: ruicao@ruc.edu.cn; b Department of Chemistry , Renmin University of China , Beijing 100872 , China; c College of Materials Science and Optoelectronic Technology , University of Chinese Academy of Science , Beijing 101408 , China

## Abstract

Ga^III^ chloride tetrakis(pentafluorophenyl)porphyrin (**1**) was synthesized and shown to be a highly active and stable post-transition metal-based electrocatalyst for the hydrogen evolution reaction (HER). Electrochemical and spectroscopic studies indicate that both the first and second reduction events of **1** are ligand-centered. The 2e-reduced form **1**^2–^ reacts with a proton to give Ga^III^–H species (**1**–H), which undergoes protonolysis with Brønsted acids to produce H_2_. The identification of key intermediates **1**^–^, **1**^2–^, and **1**–H leads to a catalytic cycle, which is valuable for the fundamental understanding of the HER. This study presents a rare but highly active HER electrocatalyst with unusual features, including ligand-centered electron transfer and formation of post-transition metal hydride.

## Abstract 2

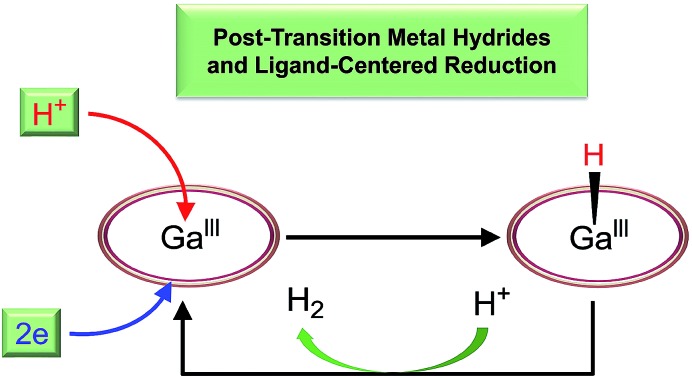
Ga^III^ porphyrin is active for electrocatalytic hydrogen evolution with unusual features, including ligand-centered electron transfer and formation of post-transition metal hydride.

## Introduction

Hydrogen is a promising energy carrier and is largely required in chemical and petroleum industries.^1^–^3^ With energy supplies from renewable sources, H_2_ can be produced through the reduction of protons in the electrolysis of water.^4^,^5^ Thus, the electrocatalytic proton reduction provides an attractive approach for producing H_2_, a clean and sustainable fuel.^1^–^3^ Extensive efforts have been made to develop molecular catalysts of earth-abundant transition metals, such as Fe,^6^–^10^ Co,^11^–^24^ Ni,^25^–^32^ and Cu,^33^,^34^ for the HER. For these transition metal catalysts, reduced metal ions are proposed to react with protons to form metal-hydride intermediates,^25^,^33^,^35^,^36^ which can then evolve H_2_ through heterolytic or homolytic pathways. Consequently, tuning both the electron and proton transfer abilities of transition metal ions is a rational strategy to improve catalytic performance.

Several ligand design strategies have been demonstrated to improve the performance of these catalysts. Multidentate phosphine ligands can stabilize low-valent metal ions and increase metal ion basicity through the π-back-donating interaction.^37^,^38^ Pendant amine groups in Ni complexes of Dubois and co-workers function as proton relays to increase activities.^39^–^41^ Similarly, appending intramolecular proton transfer sites has been shown to be successful in other catalyst systems.^42^–^46^ For these prevailing transition metal-based catalysts, metal ions are the major sites for both electron and proton transfer. As a consequence, the reduced metal ions with a low valence state usually become weaker in coordination. This will lead to possible demetalation especially under acidic conditions, which is one of the main catalyst deactivation routes in the HER.

As an alternative strategy, ligands that can mediate both electron and proton transfer have been explored recently. Catalysts of this series include Al^III^ bis(imino)pyridine,^47^–^49^ Zn^II^ thiosemicarbazone,^50^ Ni^II^ pyrazinedithiolate,^51^ Cu^II^ diacetyl-bis(*N*-4-methyl-3-thiosemicarbazonato),^52^ and Re^II^ phosphinobenzenethiolate.^53^ For these catalysts, the ligands are proposed to play essential roles in both electron transfer and proton transfer. Active X–H (X = C, N, and O) units with relatively strong covalent bond features rather than metal hydrides are considered to be involved in the H–H bond formation.

**Fig. 1:**
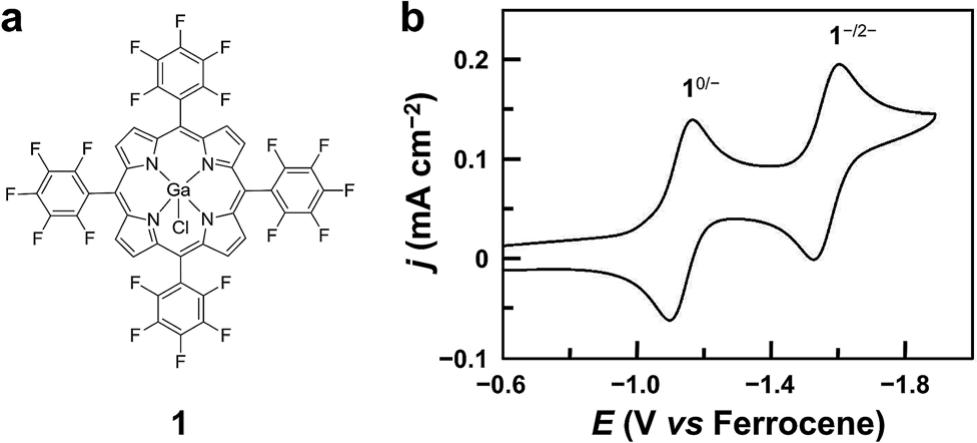
(a) Molecular structure of Ga^III^ porphyrin **1**. (b) CV of 1.0 mM **1** in acetonitrile, showing two reversible 1e reduction waves at *E*_1/2_ = –1.13 and –1.57 V. Conditions: 0.1 M Bu_4_NPF_6_, a GC working electrode, 100 mV s^–1^ scan rate, and 20 °C.

Herein, we report Ga^III^ porphyrin **1** (Fig. 1a) as a highly active and stable post-transition metal HER catalyst with the porphyrin ligand acting as the electron transfer site and the Ga^III^ ion as the hydride-binding site. Porphyrin ligands are well known to have redox-active features, which play crucial roles in several electrocatalytic processes, including the HER, and O_2_ and CO_2_ reduction reactions.^3^,^25^,^54^–^57^ Recent studies from Wang and co-workers by using a Zn porphyrin for CO_2_ reduction highlight the role of porphyrin ligands in redox catalysis.^58^ On the other hand, although Ga^III^ ions have a strong Lewis acid character and are promising in binding and transferring hydride atoms, no Ga^III^ complexes have been shown to be active for the HER. Significantly, by identifying **1**^–^, **1**^2–^, and **1**–H with spectroscopic methods and studying their reactivity, we are able to draw a catalytic cycle. Mechanistic studies provide valuable insights into the fundamental knowledge for the design of efficient post-transition metal redox catalysts.

## Results and discussion

### Synthesis and electrochemistry

Complex **1** was prepared by reacting GaCl_3_ with a free-base porphyrin in acetic acid. The details of synthesis and characterization are described in the Experimental section. The cyclic voltammogram (CV) of **1** using a glassy carbon (GC) electrode in acetonitrile with 0.1 M Bu_4_NPF_6_ displays two reversible 1e reduction waves at *E*_1/2_ = –1.13 and –1.57 V *versus* ferrocene (Fig. 1b, all potentials reported in this work are *versus* ferrocene). These two reduction waves are diffusion-controlled as suggested by the linear correlation between their peak currents and the square root of scan rates (Fig. S3†). For simplicity, the 1e- and 2e-reduced forms of **1** are denoted as **1**^–^ and **1**^2–^, respectively.

### Electrocatalytic HER studies

**Fig. 2:**
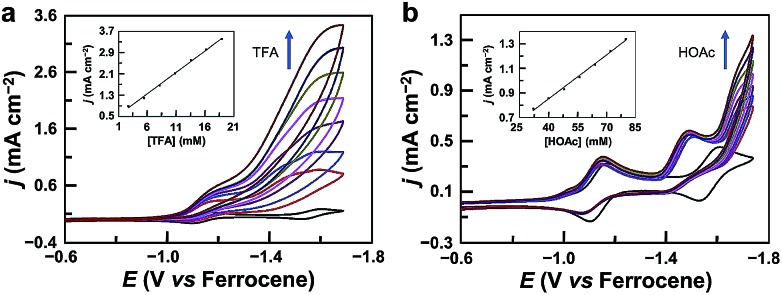
(a) CVs of 1.0 mM **1** in acetonitrile with increasing TFA. Inset: plot of catalytic peak current *versus* TFA concentration. (b) CVs of 1.0 mM **1** in acetonitrile with increasing acetic acid (HOAc). Inset: plot of catalytic current at –1.75 V *versus* HOAc concentration. Conditions: 0.1 M Bu_4_NPF_6_, a GC working electrode, and 20 °C.

The CV of **1** with the addition of trifluoroacetic acid (TFA, p*K*_a_ = 12.7 in acetonitrile^59^,^60^) shows a pronounced catalytic wave (Fig. 2a). The peak currents increase with the first-order dependence on the concentrations of both TFA (Fig. 2a, inset) and **1** (Fig. S4†), indicating catalytic proton reduction. Control experiments using GaCl_3_ (Fig. S5†) and the free-base porphyrin (Fig. S6†) gave very small currents under the same conditions. This result confirms that the catalytic activity is from **1** rather than from the demetalated species. It is necessary to note that this free-base porphyrin is active for the electrocatalytic HER if a stronger *p*-toluenesulfonic acid is used as the proton source with much more negative potentials applied.^61^ The HER activity of **1** reached an acid-independent region with more than 97 equivalents of TFA (Fig. S7†). The catalytic current *i*_cat_ value showed a scan rate *ν*-independence with *ν* > 1.0 V s^–1^ (Fig. S8†), and the *i*_cat_/*i*_p_ value (*i*_p_ is the peak current of the first reduction wave) also displayed an inflection point at *ν* = 1.0 V s^–1^ (Fig. S9†). Therefore, under these pure kinetic conditions, we can estimate the turnover frequency (TOF) of **1** for the HER to be up to 9.4 × 10^4^ s^–1^ using the reported foot of the wave analysis (FOWA, Fig. S10†).^62^,^63^ This value is comparable to those of transition metal-based HER catalysts,^25^,^33^,^63^–^66^ although it is obtained at relatively large overpotentials. In addition, we examined the electrocatalytic HER activity of Ga^III^ complexes of 5,15-bis(pentafluorophenyl)-10,20-diphenylporphyrin (Fig. S11†) and 5,10,15,20-tetrakisphenylporphyrin (Fig. S12†). By replacing *meso*-C_6_F_5_ groups with phenyl groups, the onset of the catalytic HER wave shifts to the negative direction by more than 370 mV.

The catalytic stability of **1** was verified by using controlled potential electrolysis. The UV-vis spectrum was first collected before electrolysis, showing that **1** remains unchanged in 50 mM TFA acetonitrile solution (Fig. S13†). Electrolysis was then conducted at –1.70 V in a three-compartment cell. During 5 h electrolysis using a 0.07 cm^2^ GC working electrode, substantial and stable currents at *ca.* 13.1 mA cm^–2^ could be maintained (Fig. S14†), and H_2_ gas bubbles were observed on the GC electrode surface. The amount of produced H_2_ was determined by gas chromatography, giving a faradaic efficiency of 97% for H_2_ generation (Fig. S15†). The turnover number (TON) was 18 with respect to the total amount of catalyst in the solution. As a control, electrolysis with the same concentration of TFA but without **1** gave much smaller currents at 0.7 mA cm^–2^. After electrolysis, the solution of **1** showed a UV-vis spectrum almost identical to that before electrolysis (Fig. S16†). The GC electrode after electrolysis showed almost the same current as a freshly cleaned GC electrode did in a TFA solution without **1** (Fig. S17†). In addition, surface analysis by scanning electron microscopy and energy-dispersive X-ray spectroscopy (Fig. S18†) excluded the formation of any heterogeneous phase on the GC electrode.

We also examined the proton reduction activity of **1** by using weak acetic acid (p*K*_a_ = 22.3 in acetonitrile^59^,^60^). As shown in Fig. 2b and S19,† in the presence of acetic acid, the first redox couple shows a tiny decrease in the reverse oxidation wave, while the second redox couple completely loses the reversibility in the reverse oxidation scan. This result indicates that **1**^2–^ is the active species for proton reduction to form Ga^III^–H species. Significantly, the second reduction peak of **1** still exists, and the catalytic wave appears well behind the second reduction wave, indicating that Ga^III^–H needs to be further reduced to drive the catalysis with acetic acid for H_2_ generation.

The different electrochemical behaviors of **1** with TFA and acetic acid are likely due to the difference in reactivity of **1**–H with the two acids. We can get insights from the thermodynamic analysis. As shown in eqn (1)–(7), the driving force for the **1**^2–/0^ redox couples to reduce TFA to generate H_2_ is 0.57 V, but this value is only 0.01 V to reduce acetic acid to produce H_2_. This result is consistent with the observation that more negative potentials are required to drive the catalysis using acetic acid. Notably, the value of –0.028 V was used for calculating the standard potential of the solvated H^+^/H_2_ couple in acetonitrile.^67^ If the value of –0.14 V was used,^59^ then the driving forces for the **1**^2–/0^ redox couples to reduce TFA and acetic acid to produce H_2_ were 0.45 and –0.11 V, respectively.

1 **1**^–^ → **1** + e^–^, –*E*° = 1.13 V

2 **1**^2–^ → **1**^–^ + e^–^, –*E*° = 1.57 V

3 **1**^2–^ → **1** + 2e^–^, –*E*° = 1.35 V

4 2TFAH + 2e^–^ → H_2_ + 2TFA^–^, *E*° = –0.78 V

5 2HOAc + 2e^–^ → H_2_ + 2OAc^–^, *E*° = –1.34 V

6 **1**^2–^ + 2TFAH → **1** + H_2_ + 2TFA^–^, Δ*E*° = 0.57 V

7 **1**^2–^ + 2HOAc → **1** + H_2_ + 2OAc^–^, Δ*E*° = 0.01 V

In the case of acetic acid as the proton source, the *i*_cat_ at –1.75 V increases with the first-order dependence on the concentrations of both acetic acid (Fig. 2b, inset) and **1** (Fig. S20†), and it shows *ν*-independence when *ν* is larger than 2.5 V s^–1^ (Fig. S21†), indicating catalytic proton reduction. Electrolysis of **1** with acetic acid was also performed. During 5 h electrolysis at –1.70 V using a 1.0 cm^2^ GC working electrode, a current of *ca.* 0.8 mA cm^–2^ could be maintained (Fig. S22†) with a TON of 14 and a faradaic efficiency of 96% for H_2_ generation (Fig. S23†). The stability of **1** during the electrolysis with acetic acid was also confirmed: the UV-vis spectrum showed negligible changes after electrolysis (Fig. S24†); the GC electrode after electrolysis gave no catalytic current in acetic acid solution without **1** (Fig. S25†); surface analysis also excluded the formation of any heterogeneous phase on the GC electrode.

### Mechanism studies

**Fig. 3:**
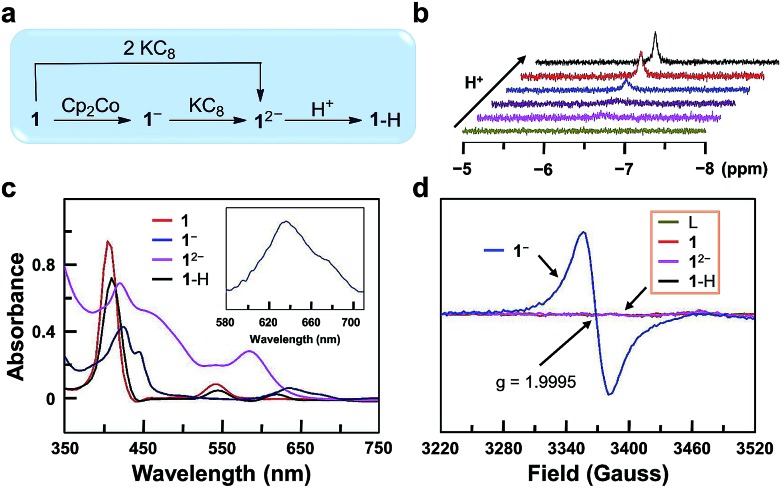
(a) Synthetic route to **1**^–^, **1**^2–^, and **1**–H. (b) ^1^H NMR spectra for the formation of **1**–H in CDCl_3_ with increasing HOAc. The Ga^III^–H peak is found at –6.45 ppm. (c) UV-vis spectra of **1**, **1**^–^, **1**^2–^, and **1**–H. (d) EPR spectra of the free-base porphyrin (L), **1**, **1**^–^, **1**^2–^, and **1**–H.

In order to get more insights into the reaction mechanism, we synthesized and characterized **1**^–^, **1**^2–^, and **1**–H (Fig. 3a). Reduction of **1** with one equivalent of cobaltocene (Cp_2_Co) gave **1**^–^ under a N_2_ atmosphere. The CV of Cp_2_Co is depicted in Fig. S26,† showing a reversible 1e reduction wave at –1.33 V. This value is between the two reduction waves of **1**. Further reduction of **1**^–^ by one equivalent of potassium graphite (KC_8_, *E*_p,c_ < –2.0 V) leads to **1**^2–^. Moreover, **1**^2–^ can be prepared separately from **1** with two equivalents of KC_8_. Reaction of **1**^2–^ with one equivalent of acetic acid leads to **1**–H. This reaction can be monitored by ^1^H NMR, showing the building up of the hydride signal at –6.45 ppm with increasing acetic acid concentration (Fig. 3b). The substantial upfield shift of this Ga^III^–H signal by more than 13 ppm compared to the reported Ga^III^–H signals^68^,^69^ is consistent with its location at the center of the aromatic porphyrin macrocycle. Notably, these ^1^H NMR data exclude the formation of phlorin species, which can possibly be generated by the protonation at the *meso*-position of porphyrin units and have typical chemical shifts of the *meso*-H at 3–5 ppm.^70^

UV-vis spectroscopic studies confirm this reaction sequence (Fig. 3c). Upon reduction, the absorption in the 560–700 nm range increases significantly. This indicates ligand-centered reduction to give 1e-reduced porphyrin anion radical **1**^–^ and 2e-reduced porphyrin dianion **1**^2–^. After the reaction of **1**^2–^ with acids, the resulting complex **1**–H shows weak absorption in the 560–700 nm range, which suggests the reduction of a proton by **1**^2–^. Notably, the UV-vis spectrum of **1**–H further confirms the protonation at the Ga^III^ ion rather than at the *meso*-position of porphyrin, since for the latter case, the resulting phlorin species will have the characteristic very broad and intense absorption in the range of 650–800 nm.^71^

Electron paramagnetic resonance (EPR) analysis provides additional evidence for the ligand-centered reduction processes. Complexes **1**, **1**^2–^, **1**–H and the free-base porphyrin are EPR-silent at room temperature (Fig. 3d). Complex **1**^–^ shows a single signal at *g* = 1.9995. This result suggests a ligand-centered reduction, because hyperfine splitting signals, which are indicators of Ga^II^ (*I* = 3/2, 4s^1^), are not observed in the EPR spectrum of **1**^–^. Furthermore, we synthesized the Ga^III^ complex of 1,4,7,10-tetraazacyclotetradecane (Fig. S27†). The CV of this Ga^III^ complex with a redox-inactive ligand does not show any reduction wave under the same conditions by scanning to –1.95 V (Fig. S28†). In addition, the CV of the free-base porphyrin displayed two reversible 1e reduction waves at *E*_1/2_ = –1.17 and –1.63 V (Fig. S29†), which are close to those of complex **1** (–1.13 and –1.57 V). Based on these results, we can conclude that the two reduction events are both ligand-centered.

**Fig. 4:**
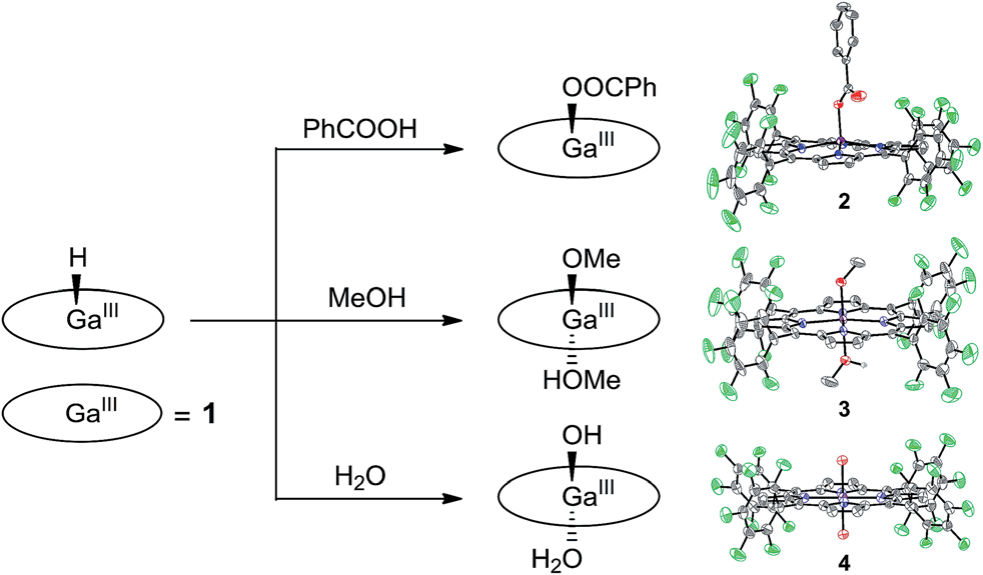
Schematic representation showing the reaction of **1**–H with different Brønsted acids and the corresponding products with thermal ellipsoid plots (50% probability) of their X-ray structures.

Attempts to obtain high quality crystals of **1**–H suitable for X-ray analysis were not successful. As an alternative, the reactions of **1**–H with different Brønsted acids were studied, and the products were structurally characterized, providing further evidence for this Ga^III^–H hydride species. The reaction of **1**–H with excess benzoic acid in acetonitrile gives **2**, which contains a benzoate at the axial position of Ga^III^ with a short Ga–O bond length of 1.872(2) Å (Fig. 4).

Crystal growth of **1**–H in methanol produces **3**, having the two axial positions occupied by a methoxide anion with a short Ga–O bond length of 1.882(3) Å and a methanol molecule with a long Ga–O bond length of 2.085(3) Å. The reaction of **1**–H with excess water gives **4**. The Ga atom in **4** is located at the crystallographically required inversion center, leading to the symmetry equivalence of the two axial O atoms with a Ga–O bond length of 1.9827(12) Å. Notably, Mayer and co-workers reported the Ga–OH and Ga–OH_2_ bond lengths of Ga^III^ porphyrin analogues as 1.810(8) and 2.091(3) Å, respectively.^72^ The Ga–O bond length as observed in **4** is exactly at the middle of these values. Therefore, we assign the two axial groups as one hydroxide and one aqua group, which are positionally disordered due to the crystallographically imposed symmetry. All these results are consistent with the protonolysis of Ga^III^–H with Brønsted acids to produce H_2_ and the corresponding Ga^III^–OR species (R = PhCO for **2**, Me for **3**, and H for **4**).

It is necessary to note that although water and methanol are weaker acids than acetic acid, their reactions with Ga^III^–H are still observed. This can be explained by the following two reasons. First, water and methanol are used in large excess, and methanol is even used as the solvent. Consequently, the equilibrium will be moved to the product’s direction. Second, the thermodynamic analysis is only used for estimating the reaction of **1**^2–^ + 2ROH → **1** + H_2_ + 2RO^–^. However, the reaction for the formation of **3** and **4** is **1**–H + ROH → **1**–OR + H_2_. The binding of the methoxide anion and hydroxide anion to Ga^III^ will certainly lower the energy of the product, which leads to different thermodynamic results. Moreover, the reaction of Ga^III^–H with water and methanol evolves H_2_, which is an entropy-increased process. In addition, this process happens on a long time scale (*i.e.*, in days) during the growth of crystals. These factors together may explain the observation of complexes **3** and **4** in this study.

**Fig. 5:**
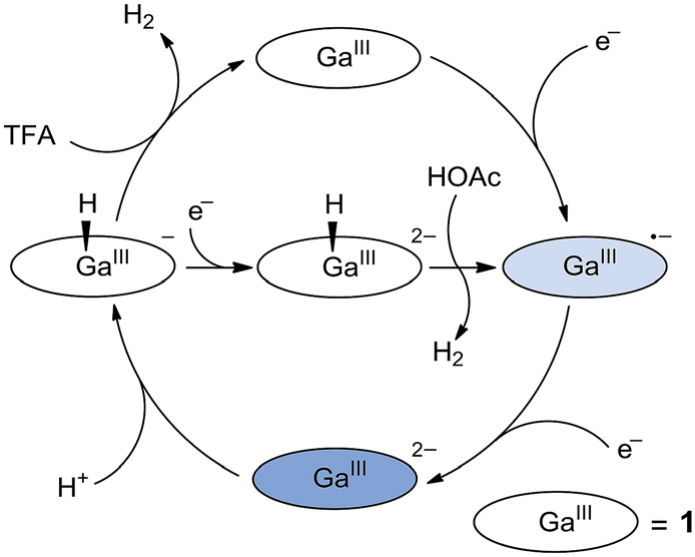
Proposed catalytic cycles for the HER by **1** with the use of either TFA or acetic acid as the proton source.

On the basis of these studies, we can draw a catalytic cycle for the HER with **1**. As shown in Fig. 5, Ga^III^ porphyrin dianion **1**^2–^ is generated upon 2e reduction of **1**. It then reacts with a proton to form the Ga^III^–H intermediate. Under electrocatalytic conditions, this hydride can undergo rapid protonolysis with a strong acid like TFA to drive the catalysis for the HER, or it needs to be further reduced by one electron to drive the catalysis with weak acetic acid. Importantly, the reaction of Ga^III^–H and deuterated TFA or acetic acid gave HD, which was detected by gas chromatography–mass spectrometry (Fig. S30†). The reaction with TFA completed instantly upon the addition of the acid (Fig. 3c), while the reaction with acetic acid completed on a much longer time scale (Fig. S31†). This difference is consistent with the relatively larger driving force of the protonolysis with TFA than that with acetic acid.

According to this mechanism, we reanalyzed the CV of **1** with TFA. As shown in Fig. 2a, when the concentration of TFA is low, there are actually two plateaus at *ca.* –1.48 and –1.62 V in the catalytic wave. This result indicates the involvement of two catalytic mechanisms. We compared the CVs of **1** with low-concentration TFA and with acetic acid (Fig. S32†). The first plateau potential as observed in the CV of **1** with TFA is similar to the second reduction wave potential of **1** in the presence of acetic acid. The second plateau potential in the CV of **1** with TFA is close to the catalytic wave of **1** with acetic acid. Importantly, with one equivalent of TFA, we can see a quasi-reversible redox couple at *E*_1/2_ = –1.66 V (Fig. S33†). As we demonstrated above, after 2e reduction, **1**^2–^ can react with a proton to generate Ga^III^–H. This leads to the proposal that this redox couple is due to the Ga^III^–H intermediate. Based on these results, we propose that at the first plateau, the generated Ga^III^–H intermediate will drive the catalysis with TFA to evolve H_2_. At more negative potentials, the Ga^III^–H intermediate can be further reduced by one electron and can then drive the catalysis with TFA, leading to the observation of the second plateau.

Very few post-transition metal complexes are demonstrated as HER catalysts. Recently, Crabtree and co-workers reported Sb^V^ porphyrins for the electrocatalytic HER.^73^ Comparison of the HER mechanisms of **1** and Sb^V^ porphyrins shows three differences. First, ligand-centered reduction is suggested in the HER with **1**, but both the porphyrin ligand and the Sb ion are redox active for Sb porphyrins. Second, **1** is the real HER catalyst, but the Sb^III^ form converted from starting Sb^V^ porphyrins is the real catalyst. Third, Ga^III^–H can react with TFA in acetonitrile to produce H_2_, while Sb^V^–H needs to be further reduced to undergo catalytic protonolysis with TFA. This difference in reactivity is likely due to the fact that Ga has a smaller electronegativity than Sb, and thus Ga–H is a stronger hydride donor than Sb–H.^74^,^75^ These differences are notable, highlighting the significance of post-transition metals in controlling the activity and mechanism of redox catalysis.

## Conclusions

In conclusion, we reported Ga^III^ porphyrin **1** as a highly active and stable post-transition metal-based electrocatalyst for the HER. On the basis of the mechanistic studies, we are able to draw a catalytic proton reduction cycle with **1**. Identifying the key intermediates **1**^–^, **1**^2–^, and **1**–H suggests that the porphyrin ligand acts as the electron transfer site and the Ga^III^ ion acts as the hydride-binding site. This ligand-centered reduction feature enables redox-inert metal ions to become catalytically active in redox reactions, and more importantly, provides a simple but general route for tuning the catalytic activity by modifying ligand substituents. This work presents the potential uses of post-transition metal complexes in redox catalysis and provides insights into the fundamental knowledge for designing efficient post-transition metal-based catalysts.

## Experimental section

### General materials and methods

All reagents were purchased from commercial suppliers and were used as received unless otherwise noted. Acetonitrile was dried by distillation with calcium hydride. Porphyrin ligands were prepared according to the methods reported previously.^25^ Tetrabutylammonium hexafluorophosphate (Bu_4_NPF_6_) was recrystallized from absolute ethanol. ^1^H NMR spectra were acquired on a Brüker spectrometer operating at 400 MHz. UV-vis absorption spectra were measured on a Hitachi U-3310 spectrophotometer. High-resolution mass spectra (HRMS) were acquired using a Brüker MAXIS. The isotopically labelled HD gas was detected by gas chromatography–mass spectrometry using a Micromeritics AutoChem-2920. X-band continuous wave electron paramagnetic resonance (EPR) measurements were carried out on a Bruker E500 EPR spectrometer at a microwave frequency of 9.45 GHz. The EPR spectrum was recorded at 298 K. The H_2_ produced during the controlled potential electrolysis was determined by using an SP-6890 gas chromatograph.

### Synthesis of Ga^III^ porphyrin **1**

The free-base porphyrin 5,10,15,20-tetrakis(pentafluorophenyl)porphyrin (300 mg, 0.31 mmol), sodium acetate (80 mg, 0.98 mmol) and excess Ga^III^ chloride (546 mg, 3.1 mmol) were added into a flask with 40 mL of dry acetic acid under a N_2_ atmosphere. The mixture was refluxed overnight, and the solvent was then removed using a rotary evaporator. The purple-red solid of **1** was acquired by silica gel column chromatography (CH_2_Cl_2_/CH_3_OH = 10 : 1 v/v) with a yield of 83.8%. ^1^H NMR (400 MHz, CDCl_3_): *δ* = 9.16 (s, 8H) (Fig. S1†). High-resolution ESI-MS for [C_44_H_8_F_20_N_4_Ga]^+^: calcd 1040.9685; found, 1040.9671 (Fig. S2†). Anal. calcd for [GaCl(C_44_H_8_F_20_N_4_)]: C, 49.04; H, 0.75; N, 5.20. Found: C, 49.23; H, 0.81; N, 5.32.

### Synthesis of Ga^III^ chloride 5,15-bis(pentafluorophenyl)-10,20-diphenylporphyrin

The synthetic procedure of this Ga^III^ porphyrin is identical to that of **1** except for the use of 5,15-bis(pentafluorophenyl)-10,20-diphenylporphyrin. ^1^H NMR (400 MHz, CDCl_3_): *δ* = 9.17 (d, *J* = 5.2 Hz, 4H), 9.00 (d, *J* = 5.2 Hz, 4H), 8.25 (dd, *J* = 7.8 Hz, 1.4 Hz, 4H), 7.70–7.90 (m, 6H). High-resolution ESI-MS for [C_44_H_18_F_10_N_4_Ga]^+^: calcd 861.0627; found, 861.0630.

### Synthesis of Ga^III^ chloride 5,10,15,20-tetrakisphenylporphyrin

The synthetic procedure of this Ga^III^ porphyrin is identical to that of **1** except for the use of 5,10,15,20-tetrakisphenylporphyrin. ^1^H NMR (400 MHz, CDCl_3_): *δ* = 9.05 (s, 8H), 8.11 (dd, *J* = 7.8 Hz, 1.4 Hz, 8H), 7.90–8.04 (m, 12H). High-resolution ESI-MS for [C_44_H_28_N_4_Ga]^+^: calcd 681.1569; found, 681.1575.

### Synthesis of Ga^III^ chloride 1,4,7,10-tetraazacyclotetradecane

Excess Ga^III^ chloride (100 mg, 0.58 mmol) and 1,4,7,10-tetraazacyclotetradecane (510 mg, 2.9 mmol) were added to acetonitrile under a N_2_ atmosphere. The mixture was refluxed overnight, and the solvent acetonitrile was then removed using a rotary evaporator. The product was acquired by slow vapor diffusion of ethyl ether to an acetonitrile solution of this Ga^III^ complex (yield 89.1%). High-resolution ESI-MS for [C_8_H_20_N_4_GaCl_2_]^+^: calcd 311.0320; found, 311.0313.

### Crystallographic studies

Complete data sets for **2** (CCDC ; 1855035), **3** (CCDC ; 1855036), **4** (CCDC ; 1855037), and Ga^III^ chloride 1,4,7,10-tetraazacyclotetradecane (CCDC ; 1855038) were collected. Single crystals suitable for X-ray analysis were coated with Paratone-N oil, suspended in a small fiber loop, and placed in a cooled gas stream at 153(2) K on a Bruker D8 Venture X-ray diffractometer. Diffraction intensities were measured using graphite monochromated Mo Kα radiation (*λ* = 0.71073 Å). Data collection, indexing, data reduction and final unit cell refinements were carried out using APEX2;^76^ absorption corrections were applied using the program SADABS.^77^ The structure was solved by direct methods using SHELXS^78^ and refined against *F*^2^ on all data by full-matrix least squares with SHELXL,^79^ following the established refinement strategies. In all crystal structures, all non-hydrogen atoms were refined anisotropically. All hydrogen atoms bound to carbon were included into the model at geometrically calculated positions and refined using a riding model. The isotropic displacement parameters of all hydrogen atoms were fixed to 1.2 times the *U* value of the atoms they are linked to (1.5 times for methyl groups). Details of the data quality and a summary of the residual values of the refinements are listed in Table S1.† The CheckCIF report for the structure of **4** shows one level A alert (Short Inter D···A Contact O1···O1). This indicates the presence of strong hydrogen bonding interactions between these oxygen atoms.

### Electrochemical studies

All electrochemical experiments were carried out using an electrochemical analyzer (CH Instruments, model CHI660D) at 20 °C, and the solution was bubbled with N_2_ gas for at least 30 min before analysis. Cyclic voltammograms (CVs) were acquired in 5 mL of dry acetonitrile containing 1.0 mM catalyst and 0.1 M *n*-Bu_4_NPF_6_ and using a three-compartment cell with a 0.07 cm^2^ glassy carbon (GC) electrode as the working electrode, Ag/Ag^+^ as the reference electrode, and a graphite rod as the auxiliary electrode. The working electrode was polished with α-Al_2_O_3_ of decreasing size (1.0 μm to 50 nm) and washed with distilled water and absolute ethanol. Ferrocene was used as an internal standard, and all potentials reported in this work are referenced to the ferrocenium/ferrocene (Fc^+^/Fc) couple. Notably, the reported Fc^+^/Fc potential values in acetonitrile can be converted to *versus* saturated calomel electrode (SCE) by adding +0.38 V as shown in previous studies.^80^ Addition of TFA (1.0 M solution in acetonitrile) or acetic acid (1.0 M solution in acetonitrile) was done using a microsyringe. Controlled potential electrolysis in 5 mL of acetonitrile solution containing 50 mM TFA with or without 1.0 mM **1** was measured at –1.70 V in a three-compartment cell with the same electrodes as that for CV measurements. H_2_ gas produced during the electrolysis was measured using an SP-6890 gas chromatograph.

## Conflicts of interest

There are no conflicts to declare.

## Supplementary Material

Supplementary information

Crystal structure data
