# Supplementary material for: Electrocatalytic hydrogen evolution with gallium hydride and ligand-centered reduction
Source: Chem Sci. 2018 Dec 27;10(8):2308–14. doi: 10.1039/c8sc05247f (PMC6385664; doi:10.1039/c8sc05247f)
Supplement: Supplementary file 1 [file SC-010-C8SC05247F-s001.pdf]

Electronic Supplementary Material (ESI) for Chemical Science.
This journal is © The Royal Society of Chemistry 2018

Electronic Supplementary Information

Electrocatalytic hydrogen evolution with gallium hydride and ligand-centered reduction

Ni Wang,[a] Haitao Lei,[a] Zongyao Zhang,[b] Jianfeng Li,[c] Wei Zhang,[a] and Rui Cao*[ab]

[a]*Key Laboratory of Applied Surface and Colloid Chemistry, Ministry of Education, School of Chemistry and Chemical Engineering, Shaanxi Normal University, Xi'an 710119, China.*

[b]*Department of Chemistry, Renmin University of China, Beijing 100872, China.*

[c]*College of Materials Science and Optoelectronic Technology, University of Chinese Academy of Science, Beijing 101408, China.*

*Correspondence Email: ruicao@ruc.edu.cn

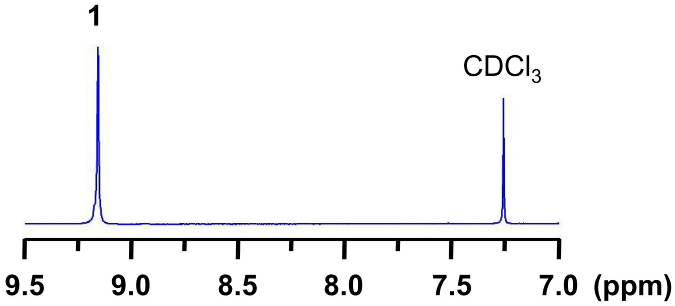

Figure S1. ^{1}H NMR spectrum of **1** in $CDCl_3$.

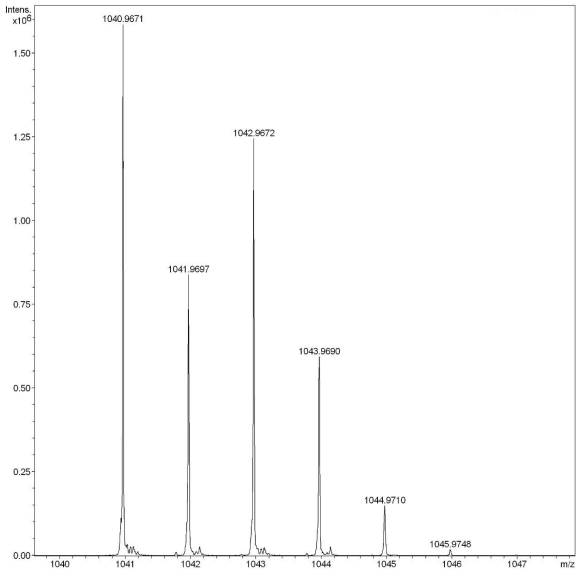

Figure S2. High-resolution mass spectrum of **1** in acetonitrile. The ion at a mass-to-charge ratio of 1040.9671 matches the calculated value of 1040.9685 for the monocation of $[C_{44}H_8F_{20}N_4Ga]^+$.

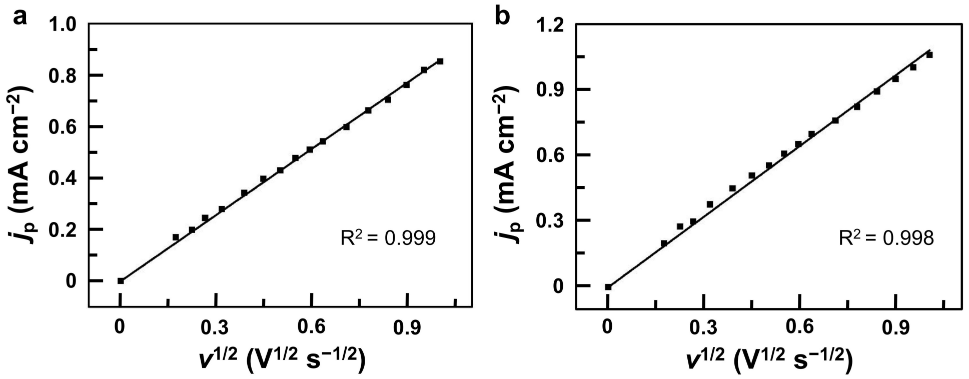

Figure S3. The peak currents of the reduction waves of **1** at $E_{1/2}$ = −1.13 (a) and −1.57 V (b) versus the square root of scan rates. The linear correlation between these two peak currents and the square root of scan rates indicated that both reduction events are diffusion-controlled.

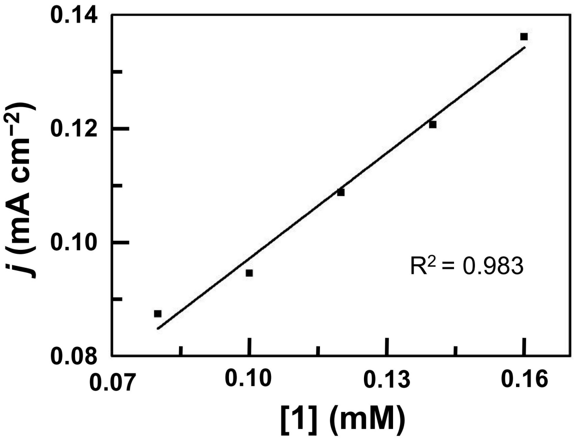

Figure S4. Plot of catalytic currents of 1 mM TFA in acetonitrile with increasing concentrations of **1**, showing a first-order dependence of the catalytic peak current on the concentration of **1**. Conditions: 0.1 M Bu_4NPF_6, GC working electrode, 100 mV s^{-1} scan rate, 20 °C.

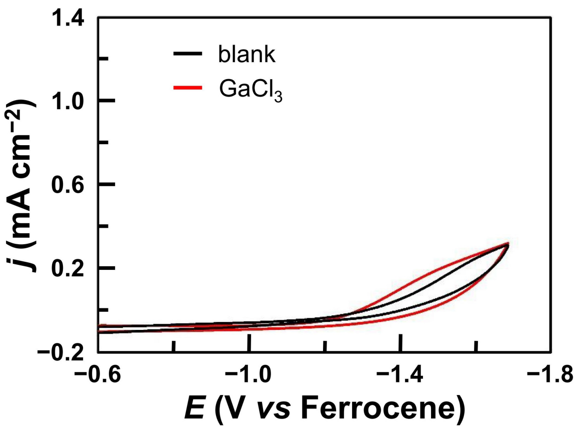

Figure S5. CVs of 15 mM TFA in acetonitrile with and without 1.0 mM $GaCl_3$. This result confirms that free Ga^{III} ions are not active for HER. Conditions: 0.1 M Bu_4NPF_6, GC working electrode, 100 mV s^{-1} scan rate, 20 °C.

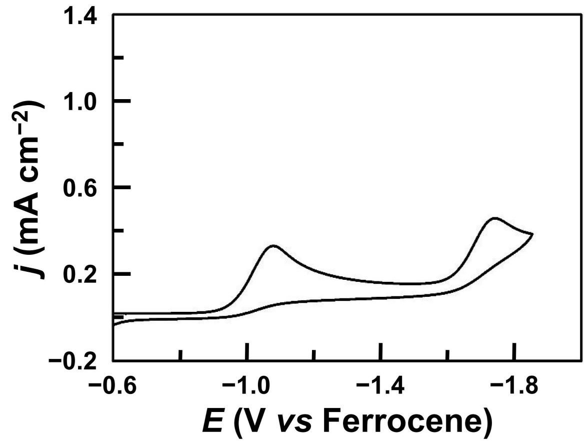

Figure S6. CV of 15 mM TFA in acetonitrile with 1.0 mM the free-base porphyrin, tetrakis(pentafluorophenyl)porphyrin. Conditions: 0.1 M Bu_4NPF_6, GC working electrode, 100 mV s^{-1} scan rate, 20 °C.

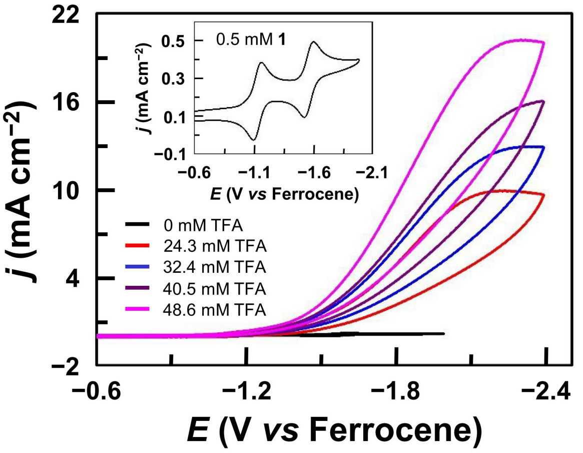

Figure S7. CVs of 0.50 mM **1** in acetonitrile with increasing TFA. Conditions: 0.1 M Bu_4NPF_6, GC working electrode, 1.0 V s^{-1} scan rate, 20 °C.

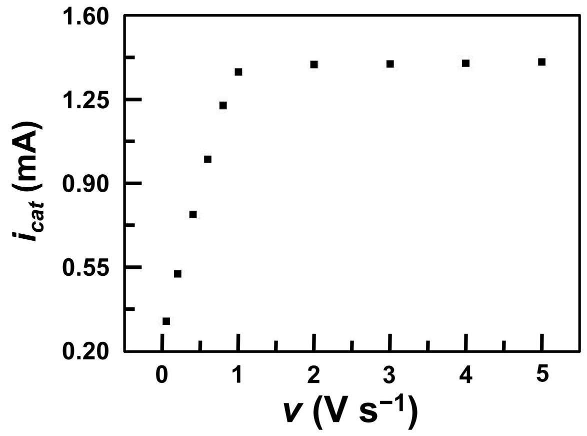

Figure S8. Dependence of the catalytic current i_{cat} on scan rate for **1**, showing that i_{cat} reaches scan rate independent at v > 1.0 V s^{-1}. Conditions: 0.1 M Bu_4NPF_6, GC working electrode, 48.6 mM TFA, 20 °C.

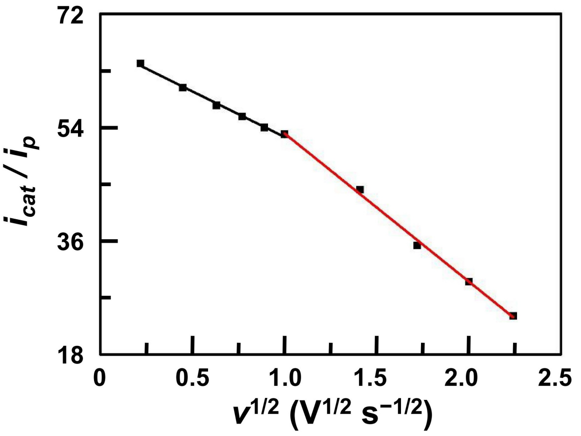

Figure S9. Dependence of i_{cat}/i_p value (i_{cat} is catalytic current; i_p is the peak current of the first reduction wave) on the square root of scan rate for **1**, showing the inflection point at 1.0 V s^{-1}. Conditions: 0.1 M Bu_4NPF_6, GC working electrode, 48.6 mM TFA, 20 °C.

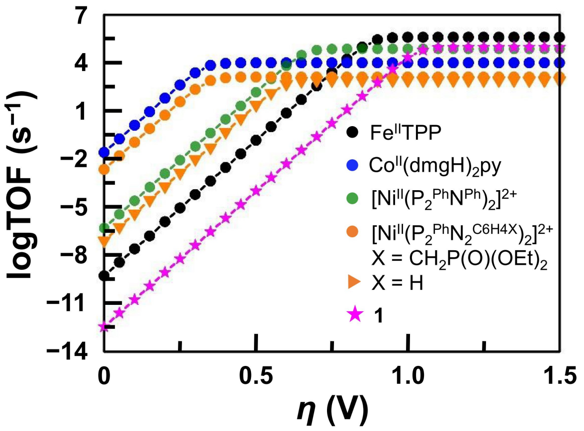

Figure S10. Catalytic Tafel plots for different electrocatalysts. Black: Fe^{II}TPP, DMF, and Et_3NH^+; Blue: $Co^{II}(dmgH)_2py$, DMF, and Et_3NH^+; Green: $[Ni(P_2^{Ph}N^{Ph})_2]^{2+}$, MeCN, and $DMFH^+$; Orange: $[Ni^{II}(P_2^{Ph}N_2^{C6H4X})_2]^{2+}$ and MeCN, X = $CH_2P(O)(OEt)_2$, $DMFH^+$ (dots), X = H, TfOH (triangles); Magenta: **1**, MeCN and TFA.

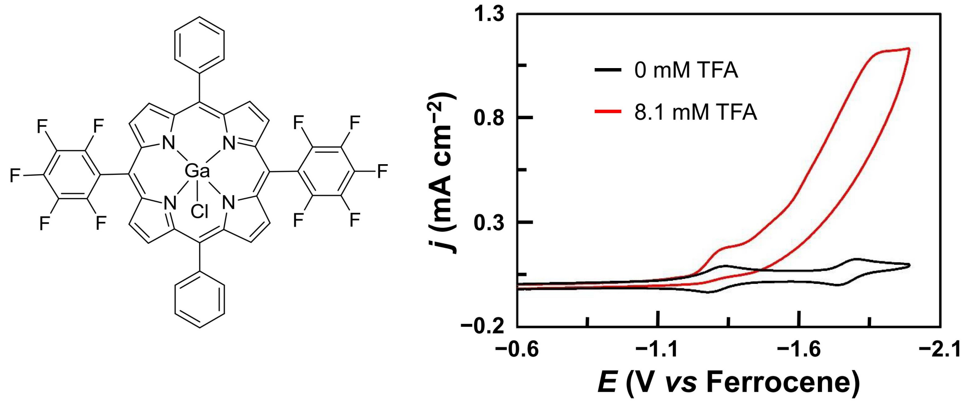

Figure S11. Molecular structure of Ga^{III} chloride 5,15-bis(pentafluorophenyl)-10,20-diphenylporphyrin (left) and its CV in acetonitrile with and without TFA (right). Conditions: 0.5 mM Ga^{III} complex, 0.1 M Bu_4NPF_6, GC working electrode, 100 mV s^{-1} scan rate, and 20 °C.

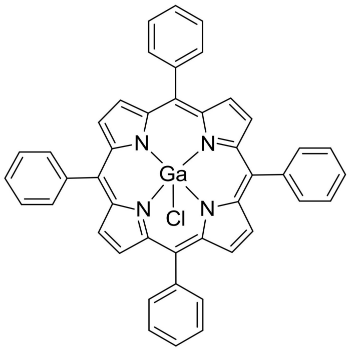

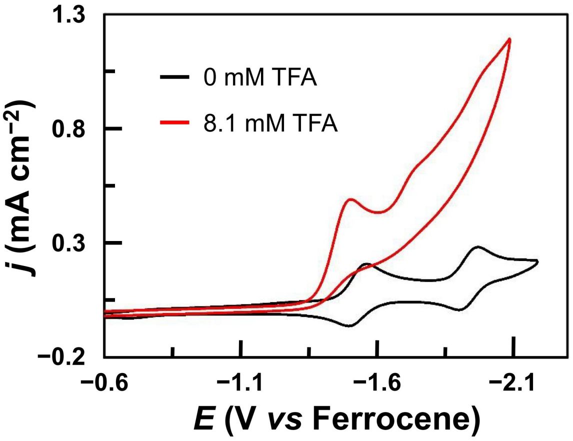

Figure S12. Molecular structure of Ga^{III} chloride 5,10,15,20-tetrakisphenylporphyrin (left) and its CV in acetonitrile with and without TFA (right). Conditions: 0.5 mM Ga^{III} complex, 0.1 M Bu_4NPF_6, GC working electrode, 100 mV s^{-1} scan rate, and 20 °C.

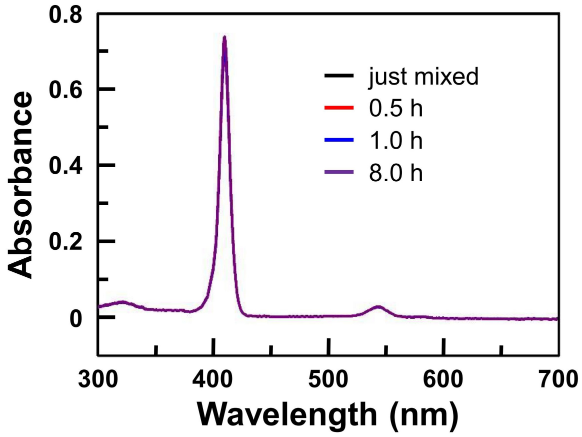

Figure S13. UV-vis spectra of **1** in the acetonitrile solution of 50 mM TFA, showing its long-term stability in strong acidic TFA solution.

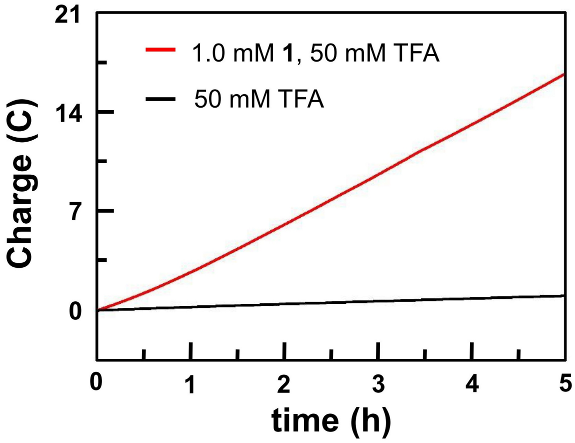

Figure S14. Charge accumulated during controlled potential electrolysis of 50 mM TFA with or without 1.0 mM **1** under an applied potential of −1.70 V using a 0.07 cm^2 GC working electrode.

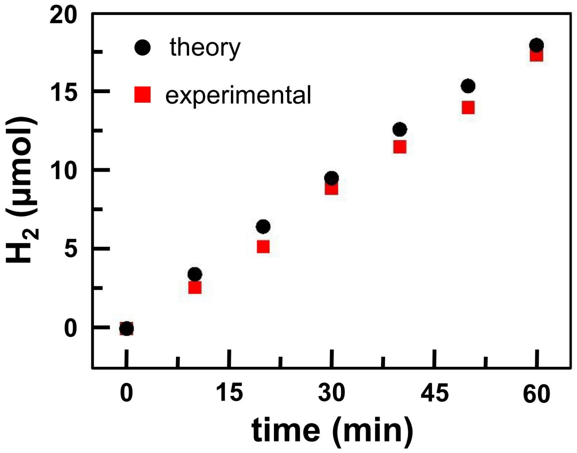

Figure S15. Gas chromatography detection of evolved H_2 during electrolysis of **1** with TFA and the theoretical amount of H_2 produced at −1.70 V, giving a Faradaic efficiency of 97% for H_2 generation.

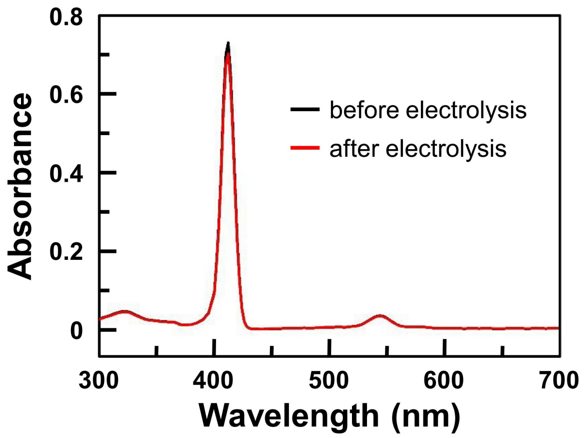

Figure S16. UV-vis spectra of **1** in acetonitrile before and after electrolysis with TFA, showing that the catalyst has sufficient stability for HER catalysis.

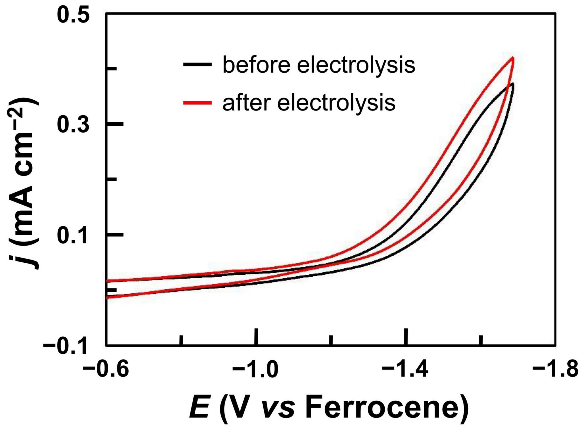

Figure S17. CVs of the GC working electrode before and after electrolysis in the TFA solution of acetonitrile, showing no deposition of active species on the surface of the GC electrode.

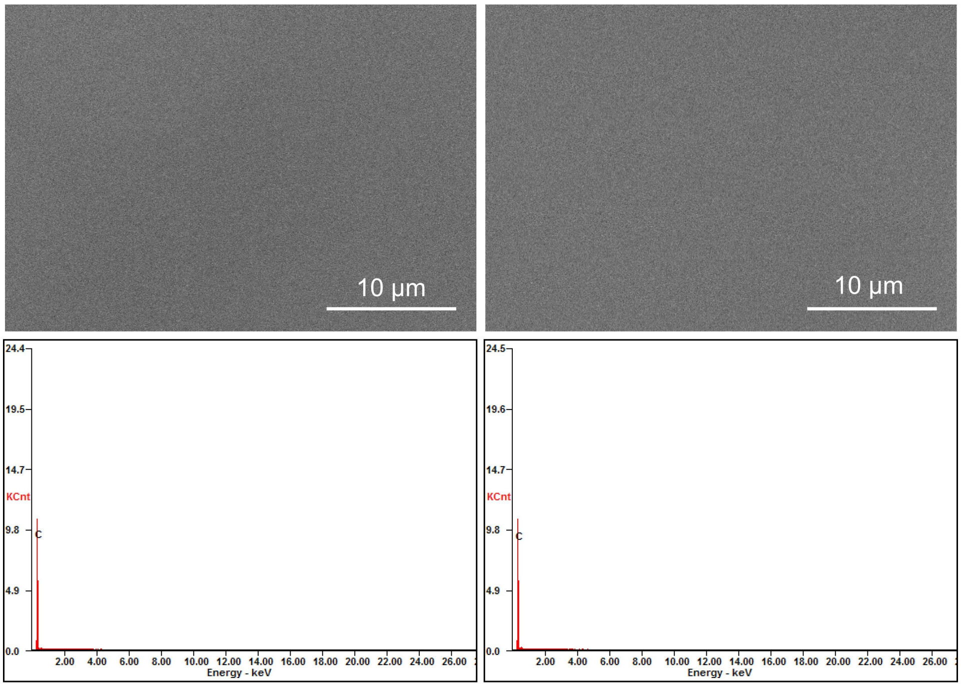

Figure S18. SEM images and EDX data of GC electrodes before (left) and after (right) electrolysis, showing no deposition of any heterogeneous phase on the surface of the GC working electrode.

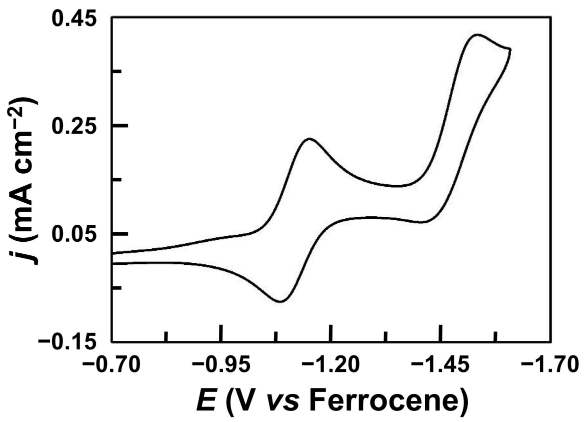

Figure S19. CV of 1.0 mM **1** in acetonitrile with 40 mM HOAc. The scan is reversed before the appearance of catalytic waves. The first redox couple shows a tiny decrease in the reversing oxidation wave, while the second redox couple completely loses the reversibility. This result indicates that $\mathbf{1}^{2-}$ is the active species for proton reduction to form the Ga^{III}–H species.

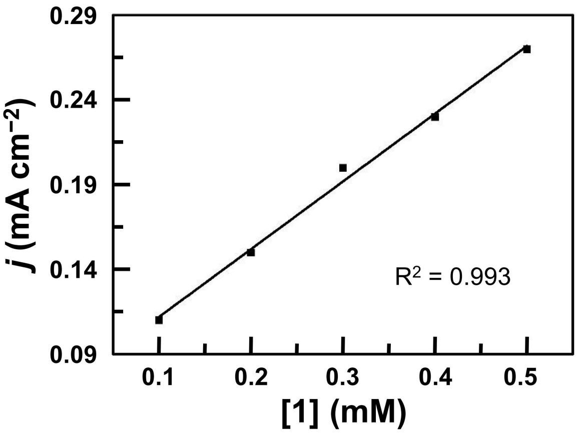

Figure S20. Plot of catalytic currents of 20 mM HOAc in acetonitrile with increasing concentrations of **1**, showing a first-order dependence of the catalytic peak current on the concentration of **1**. Conditions: 0.1 M Bu_4NPF_6, GC working electrode, 100 mV s^{-1} scan rate, 20 °C.

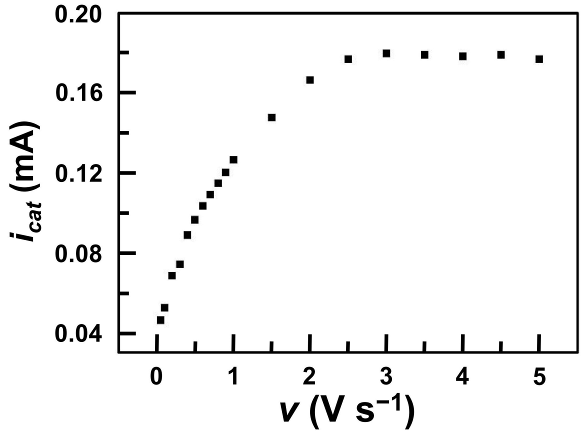

Figure S21. Dependence of the catalytic current i_{cat} on scan rate for **1**. Conditions: 0.1 M Bu_4NPF_6, GC working electrode, 80 mM HOAc, 20 °C.

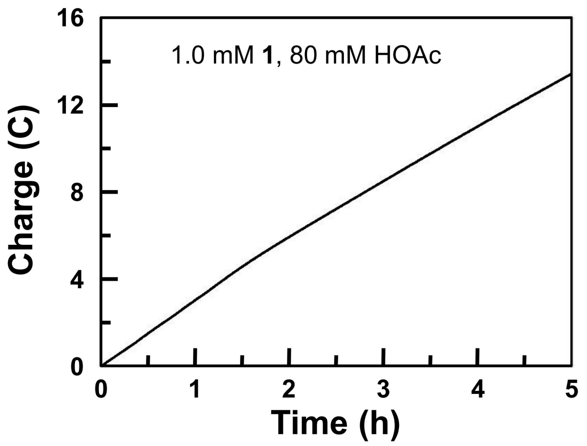

Figure S22. Charge accumulated during controlled potential electrolysis of 80 mM HOAc with 1.0 mM **1** under an applied potential of −1.70 V using a 1.0 cm^2 GC working electrode.

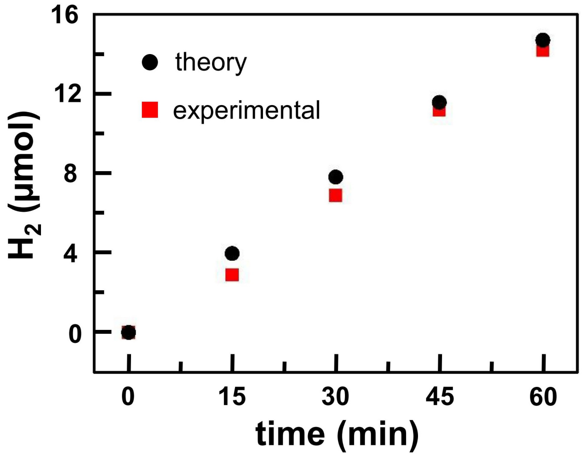

Figure S23. Gas chromatography detection of evolved H_2 during electrolysis of **1** with HOAc and the theoretical amount of H_2 produced at −1.70 V, giving a Faradaic efficiency of 96% for H_2 generation.

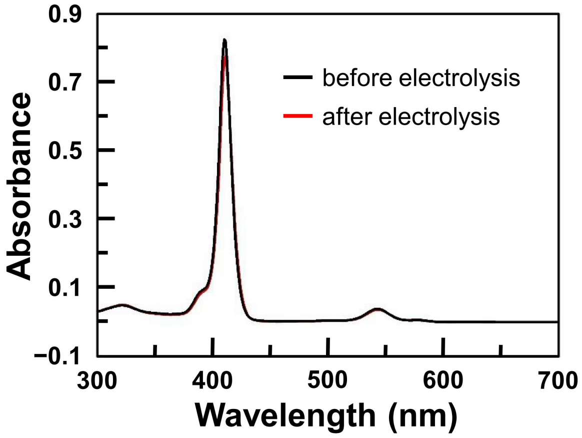

Figure S24. UV-vis spectra of **1** in acetonitrile before and after electrolysis with HOAc, showing that the catalyst has sufficient stability for HER catalysis.

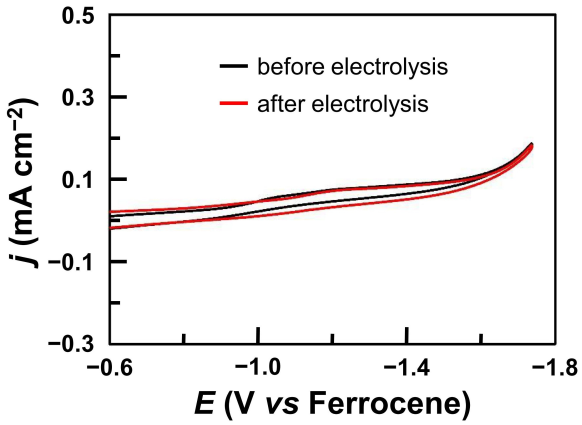

Figure S25. CVs of the GC working electrode before and after electrolysis in the HOAc solution of acetonitrile, showing no deposition of active species on the surface of the GC electrode.

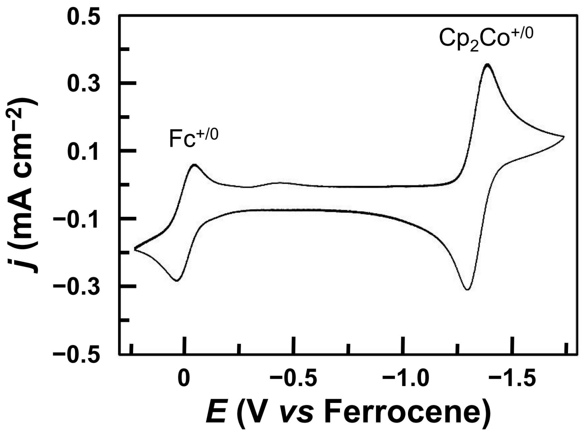

Figure S26. CV of 1.0 mM Cp_2Co in acetonitrile, showing a reversible 1e reduction wave at −1.33 V. Conditions: 0.1 M Bu_4NPF_6, GC working electrode, 100 mV s^{-1} scan rate, and 20 °C.

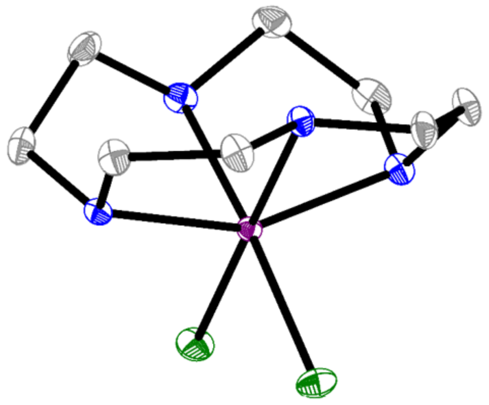

Figure S27. Thermal ellipsoid plot (50% probability) of the X-ray structure of Ga^{III} chloride 1,4,7,10-tetraazacyclotetradecane. Hydrogen atoms and the chloride counter anion are omitted for clarity.

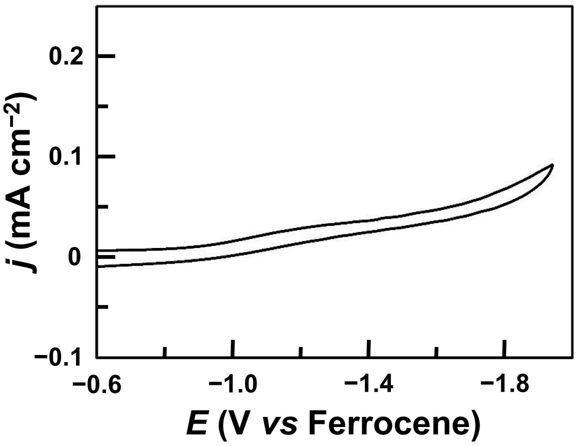

Figure S28. CV of 1.0 mM Ga^{III} chloride 1,4,7,10-tetraazacyclotetradecane in acetonitrile, showing no reduction by scanning to −1.95 V. Conditions: 0.1 M Bu_4NPF_6, GC working electrode, 100 mV s^{-1} scan rate, and 20 °C.

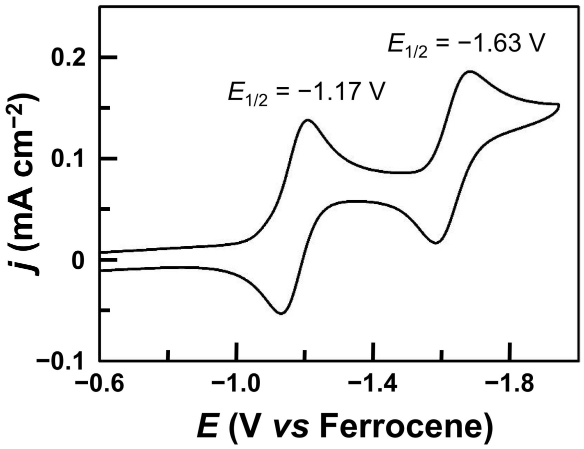

Figure S29. CV of 1.0 mM free 5,10,15,20-tetrakis(pentafluorophenyl)porphyrin in acetonitrile. These values are close to those of Ga^{III} porphyrin **1** at −1.13 and −1.57 V, further confirming the two ligand-centered reduction processes. Conditions: 0.1 M Bu_4NPF_6, GC working electrode, 100 mV s^{-1} scan rate, and 20 °C.

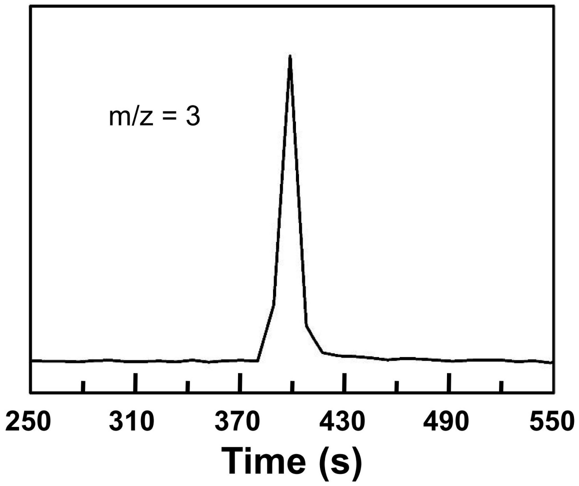

Figure S30. The gas chromatography mass spectrum of evolved HD from the reaction of **1**-H with deuterated TFA or acetic acid. The peak is at m/z = 3.

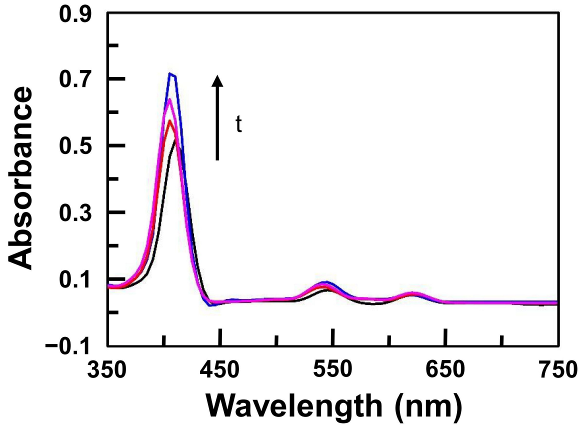

Figure S31. UV-vis spectra monitoring the reaction of **1-**H (black) and HOAc in 1 h. The last spectrum is in blue.

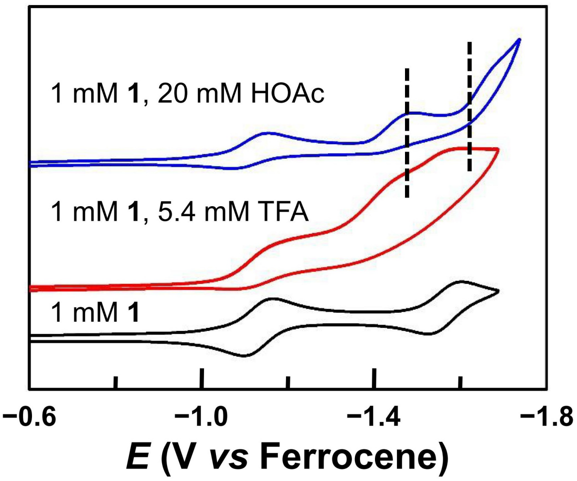

Figure S32. CVs of 1.0 mM **1** without or with TFA (5.4 mM) and HOAc (20 mM) in acetonitrile. Conditions: 0.1 M Bu_4NPF_6, GC working electrode, 20 °C.

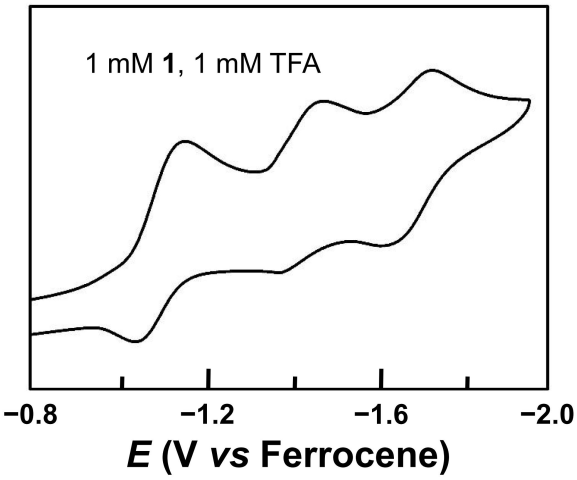

Figure S33. CV of 1.0 mM **1** with one equivalent of TFA in acetonitrile. Conditions: 0.1 M Bu_4NPF_6, GC working electrode, 20 °C.

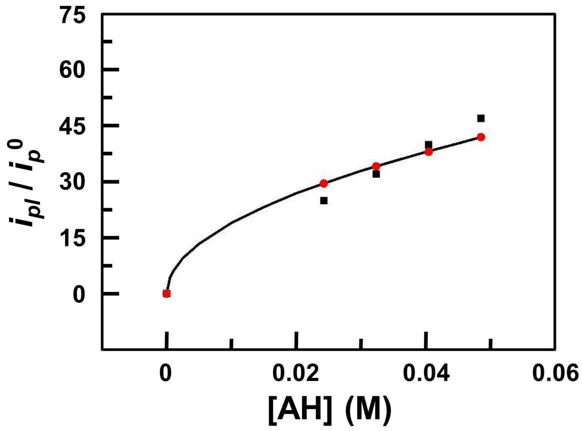

Figure S34. Variation of the normalized plateau current (solid black square) on the acid concentration and least-squares fitting (black line) of the experimental data to Equation 1 and expected normalized plateau currents (solid red circle).

This FOWA is carried out by analyzing electrocatalytic data in Figure S7 and Figure 2a using the following equations, which is established in the literature (*Energy Environ. Sci.* 2014, **7**, 3808-3814, *ACS Catal.* 2017, **7**, 3597-3606). Based on the experimental results, we can assume $k_1 >> k_2$, and then we can get $k_2 = 9.0 \times 10^4$ M^{-1} s^{-1}. Next, we use the Equation 3 to get the value of TOF_{max} at [AH] = 1 M. Finally, according to Equation 4, we can know the relationship between the TOF and overpotential.

$$E_{1/2} = E_2^0 + \frac{RT}{F}\ln\left(1+\sqrt{\frac{k_1}{k_2}}\frac{1}{1+\sqrt{\frac{k_1}{k_2}}}\right) \qquad \text{(Eq. 1)}$$

$$\frac{i_{pl}}{i_p^0} = 4.48 \frac{\sqrt{k_1[AH]}}{1+\sqrt{\frac{k_1}{k_2}}\frac{1}{1+\sqrt{\frac{k_2}{k_1}}}}\sqrt{\frac{RT}{F\nu}} \qquad \text{(Eq. 2)}$$

$$TOF_{\max} = \frac{k_1 k_2}{k_1 + k_2}[AH] \qquad \text{(Eq. 3)}$$

$$TOF = \frac{TOF_{\max}}{1+\exp\left[\frac{F}{RT}\left(E^0_{H^+/H_2} - E_{1/2}\right)\right]\exp\left(-\frac{F}{RT}\eta\right)} \qquad \text{(Eq. 4)}$$

Table S1. Crystal data and structure refinement parameters for X-ray structures **2**-**4**.

complex	**2**	**3**	**4**
molecular formula	$C_{52}H_{15}Cl_2F_{20}GaN_4O_2$	$C_{52}H_{32}F_{20}GaN_4O_5$	$C_{44}H_8F_{20}GaN_4O_2$
formula wt. (g mol^{-1})	1248.30	1242.54	1074.26
temperature (K)	153(2)	153(2)	153(2)
radiation (λ, Å)	0.71073	0.71073	1.54178
crystal system	Monoclinic	Monoclinic	Monoclinic
space group	$P2_1/c$	$P2_1/c$	$P2_1/n$
a (Å)	13.125(3)	14.3965(6)	14.0662(18)
b (Å)	17.799(3)	25.5948(10)	5.6070(8)
c (Å)	21.460(4)	13.8025(6)	26.055(3)
α (°)	90	90	90
β (°)	106.821(7)	93.4220(10)	95.056(5)
γ (°)	90	90	90
Volume (Å^3)	4798.8(16)	5076.8(4)	2047.0(5)
Z	4	4	2
ρ_{calcd} (g cm^{-3})	1.728	1.626	1.743
μ (mm^{-1})	0.812	0.669	2.190
F(000)	2464	2492	1054
crystal size (mm^3)	0.40 × 0.30 × 0.10	0.40 × 0.20 × 0.20	0.30 × 0.20 × 0.10
Theta range	2.17 to 26.46°	2.13 to 26.42°	3.41 to 68.49°
reflections collected	54038	60795	26358
indep. reflections	9852 [R(int) = 0.0727]	10408 [R(int) = 0.0784]	3752 [R(int) = 0.0474]
Completeness	99.5%	99.8%	99.6%
goodness-of-fit on F^2	1.005	1.081	1.081
final R indices [R > 2σ (I)]	$R_1^a = 0.0533$<br>$wR_2^b = 0.1143$	$R_1^a = 0.0641$<br>$wR_2^b = 0.1566$	$R_1^a = 0.0303$<br>$wR_2^b = 0.0852$
R indices (all data)	$R_1^a = 0.0769$<br>$wR_2^b = 0.1270$	$R_1^a = 0.0992$<br>$wR_2^b = 0.1796$	$R_1^a = 0.0342$<br>$wR_2^b = 0.0879$
largest diff. peak and hole (e Å^{-3})	0.797 and −0.871	0.983 and −0.603	0.620 and −0.317

$^aR_1 = \Sigma||F_o| - |F_c|| / |F_o|$, $^bwR_2 = \{\Sigma[w(F_o^2 - F_c^2)^2] / \Sigma[w(F_o^2)^2]\}^{0.5}$
